# S03-2: Lessons learned about the implementation and scalability of Fame in the UK

**DOI:** 10.1093/eurpub/ckae114.208

**Published:** 2024-09-26

**Authors:** Elizabeth Orton, Dawn Skelton, Jodi P Ventre, Fay Manning, Aseel Mahmoud

**Affiliations:** Unit of Lifespan and Population Health, University of Nottingham, Nottingham, United Kingdom; Research Centre for Health (ReaCH), School of Health and Life Sciences, Glasgow Caledonian University, Glasgow, United Kingdom; NIHR ARC-GM University of Manchester, Manchester, United Kingdom; NIHR PenARC University of Exeter, United Kingdom; NIHR PenARC University of Exeter, United Kingdom

## Abstract

Purpose: Whilst trials have shown that FaME can reduce falls and increase physical activity, it is still not routinely available across the UK. Where it is available, it is often adapted, though the impact of adaptations has not been described. The conditions necessary for FaME’s spread are not well understood. We undertook two studies that examined ‘real world' commissioning and delivery of FaME. The PhISICAl study described its effectiveness and fidelity in ethnically and geographically diverse populations, and the FLEXi study identified factors important for its spread and adoption in different commissioning landscapes, and important mechanisms for quality maintenance.

In the PhISICAl study (n = 361) FaME was associated with improvements in balance confidence (Confbal p < 0.001), fear of falling (FES-I p < 0.001), Functional Reach (p < 0.001), Turn 180o (p = 0.008) and Timed Up and Go (TUG p < 0.001) at the end of the programme versus baseline. However, improvements were not maintained at 6 months after programme completion (follow-up). Total minutes of moderate to vigorous physical activity per week was significantly higher at follow-up versus baseline (median 46 minutes, 95% CI 0-161, p = 0.047) and there was a non-significant reduction in falls at the end of FaME versus baseline (IRR 0.76, 95% CI 0.48-1.21). Up to 78% of fidelity and 84% of quality criteria were met.

In the FLEXI study (n = 596), univariate analyses showed significant improvements in Confbal (p < 0.001), falls (in the previous 3 months, p < 0.001) and TUG (p < 0.001) at programme completion vs baseline. Participants also reported wellbeing benefits and valued social opportunities. Programmes varied in duration and aspects of delivery and newer programmes or those with quality assurance systems in place showed higher fidelity and quality. A national community of practice forum was used to help improve programme fidelity and quality. Factors important for the spread and adoption of FaME included its perceived fit with decision-makers, their own personal experiences and the strength of its evidence base.

Learning has led to the development and refinement of an implementation toolkit for FaME and the establishment of a new implementation body – the National FaME Implementation Team (N-FIT) which supports the spread and implementation of FaME across the UK.

